# Review of the fish parasitic genus *Elthusa* Schioedte & Meinert, 1884 (Crustacea, Isopoda, Cymothoidae) from South Africa, including the description of three new species

**DOI:** 10.3897/zookeys.841.32364

**Published:** 2019-04-23

**Authors:** Serita van der Wal, Nico J. Smit, Kerry A. Hadfield

**Affiliations:** 1 Water Research Group, Unit for Environmental Sciences and Management, North-West University, Private Bag X6001, Potchefstroom, 2520, South Africa; 2 Zoomorphology group, Department of Biology II, Ludwig-Maximilians-University, Großhaderner Straße 2, 82152 Planegg-Martinsried, Germany

**Keywords:** Alexander Bay, Atlantic Ocean, *
Clinus superciliosus
*, *
Elthusa raynaudii
*, fish parasites, Indian Ocean, taxonomy

## Abstract

The branchial-attaching cymothoid genus, *Elthusa* Schioedte & Meinert, 1884 is a genus with a worldwide distribution of 36 species, including the three species described here. *Elthusaraynaudii* (Milne Edwards, 1840) is the only species that has been described from southern Africa. All South African material held at the National Museum of Natural History, Paris, France (MNHN) and the Iziko South African Museum, Cape Town (SAMC) identified as, or appearing to belong to, *Elthusa* was examined. Four species were identified, *Elthusaraynaudii* and three species that proved to be undescribed. *Elthusaxena***sp. n.** can be distinguished by an evenly rounded pereonite 1 anterior margin, a roughly rectangular pleotelson, and narrowly rounded uropod apices that extend to more than half the length of the pleotelson. *Elthusaacutinasa***sp. n.** is identified by the produced and narrowly rounded cephalon anterior margin, acute uropods that are shorter than half the length of the pleotelson, and pereonite 1 anterior margin with medial projection. *Elthusarotunda***sp. n.** is characterised by the round body shape, broadly rounded uropod apices, and protrusions on the proximal and lateral margins of the merus and carpus of pereopod 7. A key to the South African *Elthusa* species is provided, together with a table summarising the hosts and localities of the 33 previously known species of *Elthusa*.

## Introduction

*Elthusa* Schioedte & Meinert, 1884 is a branchial cavity-inhabiting cymothoid genus that was described as a monotypic genus for *Elthusaemarginata* (Bleeker, 1857). *Elthusa* was subsequently largely overlooked until Bruce (1990) provided a new diagnosis based on one of Bleeker’s (1857) syntypes and the Australian species of the genus. Most species of *Elthusa* were originally described and placed within the genus *Livoneca* before their revision and redescription by Bruce (1990).

Currently, there are 33 known and accepted *Elthusa* species (Öktener et al. 2018a). *Elthusa* is one of the more speciose genera within the family Cymothoidae Leach, 1818, however many species of *Elthusa* still need to be studied and redescribed due to their original descriptions being inadequate, lacking morphological detail and illustrations. The high morphological intraspecific variability that exists within this genus (Hadfield et al. 2017) has also contributed, in some cases, to misidentifications and confusion regarding the placement of species.

Most species of *Elthusa* inhabit the branchial cavities of their fish hosts (Smit et al. 2014), with the exception of two species. *Elthusaneocytta* (Avdeev, 1975) ovigerous females have been recorded from the buccal cavity of the spiky oreo, *Neocyttusrhomboidalis* Gilchrist, 1906 (see Stephenson 1987), and *Elthusasplendida* (Sadowsky & Moreira, 1981) has been described from the buccal cavity of the spiny dogfish *Squaluscubensis* Rivero, 1936 (see Sadowsky and Moreira 1981).

*Elthusa* is considered to be cosmopolitan, except for polar waters (Bruce 1990, Bruce et al. 2002, Rocha-Ramírez et al. 2005, Hadfield et al. 2017), and is predominantly recorded from the Indo-West Pacific (see Bruce 1990, Trilles and Justine 2006) with only occasional records of species from the Eastern Pacific (Brusca 1978, Espinosa-Pérez and Hendrickx 2001), the Atlantic (Kensley and Brusca 2001) and the Mediterranean (Trilles and Justine 2006, Öktener et al. 2018a). *Elthusaraynaudii* (Milne Edwards, 1840) is the only species of *Elthusa* that has been described from sub-Saharan Africa. The lack of species records is most likely due to the lack of studying cymothoid isopods from this region and is not a true representation of the biodiversity of this genus. This paper forms part of a detailed study on the cymothoids from sub-Saharan Africa and confirms this postulation with the identification of three new species from the region.

## Materials and methods

Twenty-seven specimens of *Elthusa* were examined. Material loaned from the National Museum of Natural History, Paris, France (**MNHN**) and the Iziko South African Museum, Cape Town (**SAMC**) was included in the examination. These specimens were collected as early as 1840 (MNHN) and 1960 (SAMC). Non-museum material was collected during 1993 in the intertidal zone of Alexander Bay, as well as from deep-sea trawlers during January 1999 and April 2003 off the south coast (RV Africana), and during February 2010 off the west coast of South Africa (RV Dr Fridtjof Nansen).

Specimens were identified by illustrating all body parts and appendages using a Nikon SMZ1500 Stereo Microscope and a Nikon Eclipse80i Compound Microscope, both equipped with drawing tubes. The position of specimens and dissected parts were manipulated to obtain the most accurate direct and complete view in order to minimise errors in illustrated ratios of segments. Material loaned from national museums was not dissected. Species descriptions were made with the aid of the taxonomy software package DELTA (Descriptive Language for Taxonomy) (see Coleman et al. 2010), following a general Cymothoidae character data set originally developed by Hadfield et al. (2010) and recently updated for other genera (Hadfield et al. 2013, 2016b). Ratios and measurements for the descriptions were made using the maximum values at the middle of the specific measured segment, and all proportional measurements were rounded to one decimal place. Higher-level classification follows that of Brandt and Poore (2003). Host authorities are not included in the text or references; host nomenclature and distribution were sourced from FishBase (see Froese and Pauly 2018) and Catalog of Fishes (see Eschmeyer 2018).

Abbreviations:

**DELTA** Descriptive Language for Taxonomy

**MNHN** National Museum of Natural History, Paris, France

**NWU** North-West University, Potchefstroom Campus

**OH** other hosts

**OL** other localities

**RV** research vessel

**SAMC** Iziko South African Museum

**Syn** synonym

**TH** type host

**TL** total length

**TLoc** type locality

**W** width

## Taxonomy

### Suborder Cymothoida Wägele, 1989

#### Superfamily Cymothooidea Leach, 1814

##### Family Cymothoidae Leach, 1814

###### Genus Elthusa

Taxon classificationAnimaliaIsopodaCymothoidae

Schioedte & Meinert, 1884

Elthusa : Schioedte and Meinert 1884: 337; Bruce 1990: 254; Trilles and Randall 2011: 453; Hadfield et al. 2017: 3.

####### Type species.

*Livonecaemarginata* Bleeker, 1857; by monotypy (Schioedte and Meinert 1884). The original number of type specimens that were available to Bleeker (1857) is unknown. A single female syntype, examined by Bleeker (1857), is deposited at the Naturalis Biodiversity Center (previously the Rijksmuseum von Natuurlijke Historie), Leiden (RMNH.CRUS.I.66). Another type specimen from the latter museum has been lost. The specimen examined by Schioedte and Meinert (1884) is held at the Natural History Museum in Paris (MNHN241) (Trilles 1976).

####### Remarks.

Species from *Elthusa* can be distinguished from other genera by having a weakly vaulted dorsum with a wide pleon; antennulae that are shorter than, or subequal in length to antennae, bases not in contact; a cephalon posterior margin that is not trilobed; and lamellar pleopods. Other diagnostic characters include a slender maxilliped palp article 3, with setae present; as well as pereopods with relatively short dactyli (see Bruce 1990 for a revised diagnosis of the genus).

Trilles and Randall (2011) redescribed the type species for the genus, *E.emarginata*. This redescription provided a more detailed description and more accurate drawings of the species that had previously not been possible due to the fragility of the syntype. It also allows for a diagnosis and description of the genus based on the type material. However, Trilles and Randall (2011) designated one of the examined specimens [material deposited by Schioedte and Meinert (1884) into the Natural History Museum in Paris, MNHN No. 241] as the lectotype for the species. This does not follow the ICZN rules (Article 74.1) for lectotype designation as there is extant type material (RMNH.CRUS.I.66). Furthermore, no figures were provided of the designated lectotype material to ensure recognition of the specimen designated (ICZN Article 74.7.2). As such this lectotype designation is invalid and set aside (ICZN Article 74.2).

The original description by Bleeker (1857) did not specify any host species, genus or even family (“the skin of various species of fish”) and Trilles and Randall’s (2011) redescription is not supported by or based on specimens being from the same host species or genus. Trilles and Randall (2011) did not examine Bleeker’s remaining syntype, and comparison of the two accounts suggest that there are some differences between the Bleeker (1857) figures and those of Trilles and Randall (2011); most notably being the shape of the cephalon, which is truncate or subtruncate in the syntype but anteriorly concave in Trilles and Randall’s redescription; and the pleotelson in the syntype is broadly rounded (“semi-circular”) while distally narrowed in Trilles and Randall’s redescription. Trilles and Randall (2011) made no direct reference to Bleeker’s (1857) description and did not comment on any perceived character difference. These differences suggest that direct comparison to Bleeker’s syntype is needed to confirm conspecificity of the specimens identified by Trilles and Randall (2011) as *E.emarginata*.

###### Key to the species of *Elthusa* from southern Africa

**Table:** 

1	Pleonite 5 lateral margins visible; uropods half the length of pleotelson or longer; pereonite 1 anterior margin without medial projections; pereonite 1 anterolateral margin extending to medial region of the eye	**2**
–	Pleonite 5 lateral margins largely concealed by pleonite 4; uropods short, less than half the length of pleotelson; pereonite 1 anterior margin medially pointed; pereonite 1 anterolateral margin extending to posterior margin of the eye	***Elthusaacutinasa* sp. n.**
2	Cephalon with rounded anterior margin; uropod rami apices broadly rounded; pleotelson evenly rounded	**3**
–	Cephalon anterior margin narrowly rounded; uropod rami apices narrowly rounded; pleotelson sub-quadrate	***Elthusaxena* sp. n.**
3	Pereon 1.2–1.4 times as long as wide; cephalon anterior margin blunt; pereopod 7 without bulbous protrusions; uropods more than half the length of pleotelson; pleonites subequal in length	*** Elthusa raynaudii ***
–	Pereon as long as wide; cephalon anterior margin concave; pereopod 7 merus and carpus with bulbous protrusions; uropods half the length of pleotelson; pleonite 5 longest	***Elthusarotunda* sp. n.**

###### Elthusa raynaudii

Taxon classificationAnimaliaIsopodaCymothoidae

(Milne Edwards, 1840)

**Figure 1.**
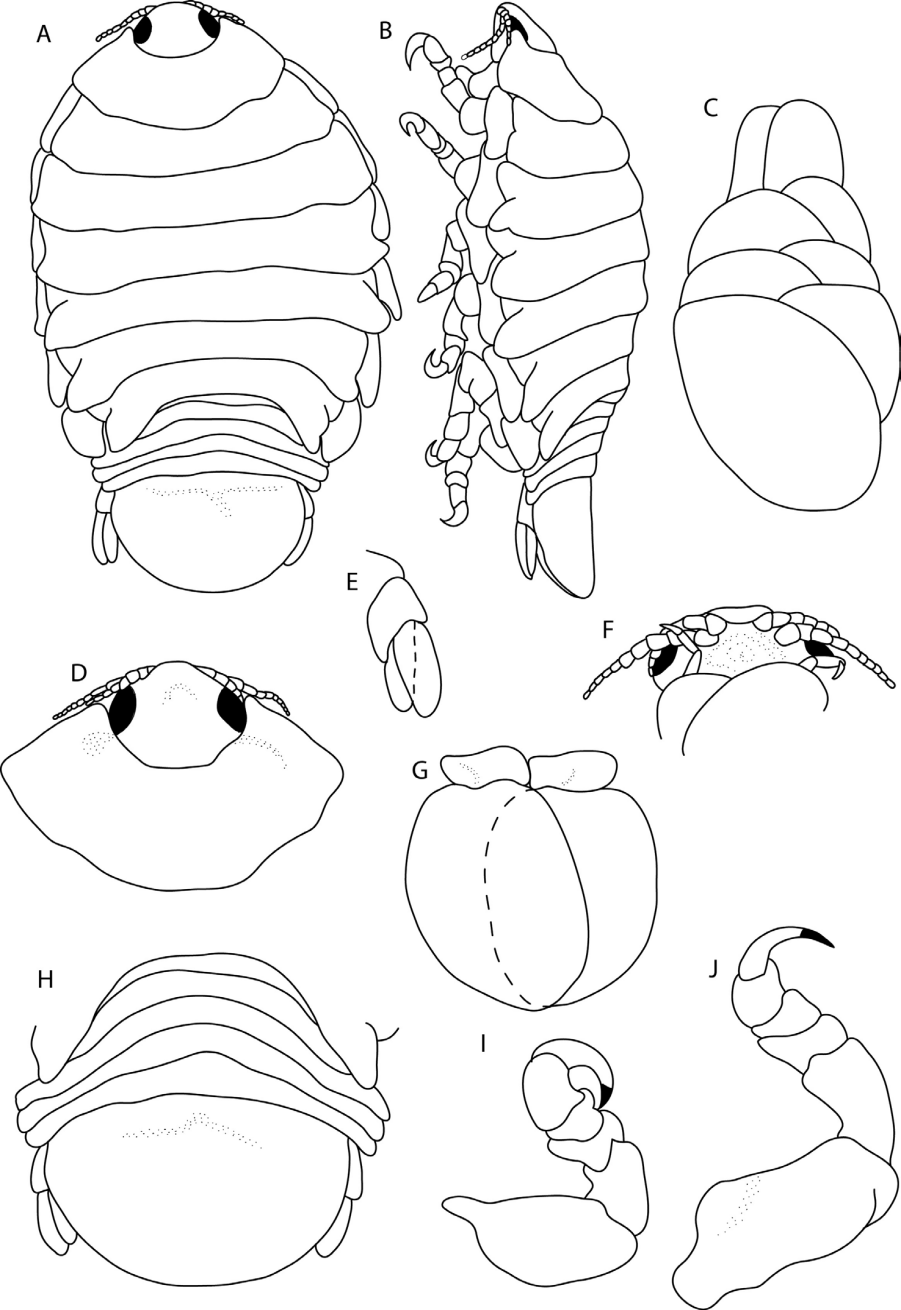
*Elthusaraynaudii* (Milne Edwards, 1840) ♀ (ovigerous, 20.0 mm TL, 12.0 mm W) (SAMC-A089957) from Dr Fridtjof Nansen research vessel **A** dorsal body **B** lateral body **C** oostegites **D** dorsal view of cephalon and pereonite 1 **E** uropod **F** ventral cephalon **G** pleopod 1 **H** dorsal view of pleon **I** pereopod 1 **J** pereopod 7.

**Figure 2.**
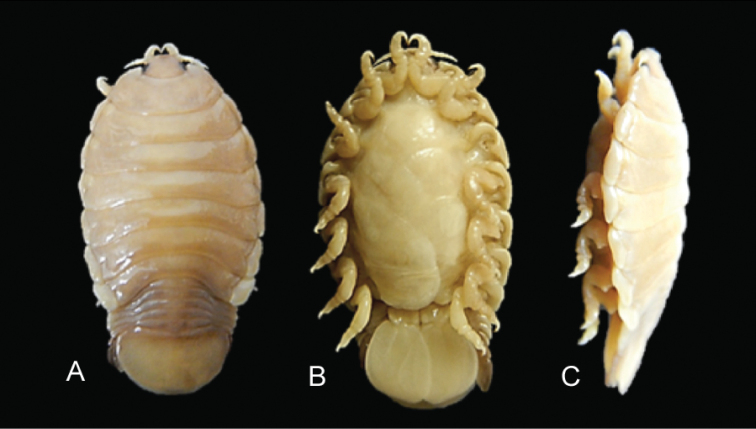
Photos of *Elthusaraynaudii* (Milne Edwards, 1840) ♀ (ovigerous, 26.0 mm TL, 15.0 mm W) (SAMC-A47881) from Dr Fridtjof Nansen research vessel **A** dorsal view **B** ventral view **C** lateral view.

**Figure 3.**
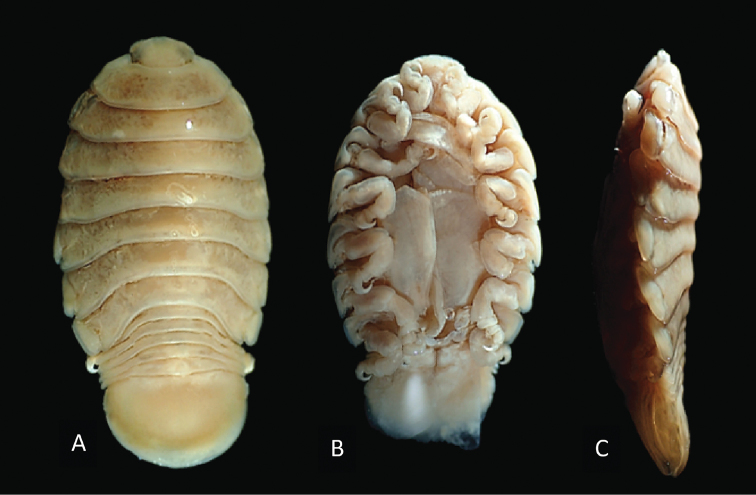
Photos of syntype material *Livonecaraynaudii* Milne Edwards, 1840 ♀ (ovigerous, 26.7 mm TL, 14.1 mm W) (MNHN–IU–2016–9885) **A** dorsal view **B** ventral view **C** lateral view.

Figures 1 , 2 , 3 , Table 1

Livoneca Raynaudii : Milne Edwards 1840: 262; Krauss 1843: 66; Bleeker 1857: 30; Schioedte and Meinert 1884: 367, pl. 12, figs 9–13; Thielemann 1910: 42; Hale 1926: 215–217, figs 10a–j.
Cymothoa Novae-Zealandia: White 1847: 110 (nomen nudum).
Lironeca novae-zealandia : Miers 1874: 228; 1876: 106, pl. III, fig. 2; 1881: 64, 67.
Lironeca laticauda : Miers 1877: 677, pl. 69, fig. 5; Ellis 1981: 124.
Livoneca Raynaudi .–Gerstaecker 1882: 259.
Livoneca Novae Zelandiae.–Gerstaecker 1882: 263.
Lironeca Stewarti: Filhol 1885: 450, pl. 4, fig. 6.
Lironeca neo-zelanica .–Thomson and Chilton 1886: 154.
Livoneca raynaudii .–Whitelegge 1902: 236; Chilton 1909: 606; 1911: 309; 1912: 135; Stebbing 1910: 125; Young 1926: 283; Hale 1926: 215, fig. 10; 1929: 261, figs 253, 259; 1940: 303; Barnard 1940: 491; 1955: 6; Hurley 1961: 268; Hewitt and Hine 1972: 108; Sivertsen and Holthuis 1980: 34; Beumer et al. 1982: 33.
Livoneca epimerias : Richardson 1909: 88, fig. 13; Kussakin 1979: 301, figs 69, 170.
Livoneca raynaudi .–Nierstrasz 1915: 97; 1931: 145; Barnard 1920: 358; Pillai 1954: 16.
Livoneca laticauda .–Nierstrasz 1931: 143.
Lironeca raynaudii .–Brian and Dartevelle 1949: 176; Avdeev 1975: 250; 1978: 281; Trilles 1976: 778, pl. 1, fig. 4; Poore 1981: 341.
Lironeca raynaudi .–Menzies 1962: 115, fig. 36A–B; Kensley 1978: 80, fig. 33B; Moreira and Sadowsky 1978: 111.
Lironeca magna : Mañé-Garzón 1979: 18, figs 1–5.
Elthusa raynaudii .–Bruce 1990: 263; Bruce et al. 2002: 177; Williams et al. 2010: 99–101.
Elthusa raynaudi .–Ghani 2003: 218.

####### Type material.

Type material held at the Museum national d’Histoire naturelle, Paris (syntypes MNHN-IU-2016-9885; MNHN-IU-2016-9884).

####### Type locality.

Cape of Good Hope, South Africa.

####### Type host.

Unknown.

####### Material examined

(all from South Africa). *Syntype*. SOUTH AFRICA • 1 ♀ (ovigerous, 26.7 mm TL, 14.1 mm W); south coast of South Africa, Cape of Good Hope; MNHN-IU-2016-9885. *Other material.* SOUTH AFRICA • 1 ♀ (ovigerous, 26.0 mm TL, 14.0 mm W); Indian Ocean, south coast of South Africa, RV Africana (fish sorting table); 34°38'S, 25°38'E; April 2003; coll. Nico J. Smit; dissected; in the collection of the authors at NWU • 1 ♀ (ovigerous, 26.0 mm TL, 15.0 mm W); Atlantic Ocean, RV Dr Fridtjof Nansen trawl (Station NAN401T062); January 2007; coll. L Atkinson; SAMC-A47881 • 1 ♀ (ovigerous, 20.0 mm TL, 12.0 mm W); Atlantic Ocean, RV Dr Fridtjof Nansen (fish sorting table); 32°17'S, 16°54'E; 269 m; February 2010; coll. KA Hadfield; dissected; SAMC-A089957.

####### Description

(ovigerous ♀). Figs 1–3. *Body* ovoid, slightly twisted to the left, 1.7 times as long as greatest width; dorsal surfaces smooth and polished in appearance, widest at pereonite 5, most narrow at pereonite 1; pereonite lateral margins mostly posteriorly ovate, medially indented. *Cephalon* 0.9 times longer than wide, visible in dorsal view, sub-truncate with blunt anterior margin. *Frontal margin* thickened, ventrally folded. *Eyes* oval with distinct margins; one eye 0.2 times width of cephalon, 0.4 times length of cephalon. *Pereonite 1* smooth; anterior border medially straight, curved laterally; anterolateral angle narrowly rounded, extending to the medial region of eyes. Posterior margins of pereonites smooth, slightly curved laterally. Coxae 2–3 wide, with posteroventral angles rounded; coxae 4–7 with rounded point, not extending past pereonite posterior margin. Pereonites 2–5 subequal, becoming more progressively rounded posteriorly; pereonites 6 and 7 slightly narrower. *Pleon* 0.4 times as long as total body length, with pleonite 1 largely concealed by pereonite 7, slightly visible in dorsal view; pleonites posterior margin mostly concave. *Pleonite 2* partially overlapped by pereonite 7. Pleonites 3–5 similar in form to pleonite 2; pleonites subequal in length, with posterolateral angles narrowly rounded, posterior margin straight. *Pleotelson* 0.6 times as long as anterior width, dorsal surface smooth; lateral margins weakly convex; posterior margin evenly rounded.

*Antennula* shorter than antenna, consisting of eight articles; antennula peduncle articles I and II distinct and articulated, extending to anterior of pereonite 1. *Antenna* consists of eleven articles, extending to middle of pereonite 1.

*Pereopod 1* basis 1.6 times as long as greatest width; ischium 0.7 times as long as basis; merus proximal margin without bulbous protrusion; carpus with rounded proximal margin; propodus 1.4 times as long as wide; dactylus slender, 1.6 times as long as propodus, 2.9 times as long as basal width. All pereopods without robust or simple setae. *Pereopod 7* basis with carina, 2.5 times as long as greatest width; ischium without protrusions, 0.5 times as long as basis; merus 0.7 times as long as wide, 0.4 times as long as ischium; carpus without bulbous protrusion, 0.7 times as long as wide, 0.3 times as long as ischium; propodus 0.8 times as long as wide, 0.3 times as long as ischium; dactylus slender, 2.3 times as long as propodus, 3.5 times as long as basal width.

*Pleopods* simple, exopod larger than endopod. *Pleopod 1* exopod 1.3 times as long as wide, lateral margin weakly convex, distally narrowly rounded, mesial margin straight; peduncle 2.3 times as wide as long.

*Uropod* more than half the length of pleotelson; peduncle 0.5 times longer than rami, lateral margin without setae; rami not extending beyond pleotelson, apices broadly rounded. *Endopod* apically rounded, 2.7 times as long as greatest width, terminating without setae. *Exopod* extending to end of endopod, 2.2 times as long as greatest width, apically rounded, terminating without setae.

*Variations.* Intra-specific variations can cause difficulty in identification and should be taken into consideration. One of the more obvious variations is the overall body shape of examined individuals, as seen from the dorsal view. While the syntype (MNHN–IU–2016–9885) has weakly convex, symmetrical lateral margins, specimen SAMC-A089957 is not as symmetrical, with the right margin being strongly convex and that of the left margin, weakly convex. The latter specimen therefore appears to be less symmetrical. Bruce (1990) mentioned this occasional asymmetrical body shape as an observed variation, as a result of slightly twisted individuals. The body shape of the South African specimen (SAMC-A089957) accords to the shape of individuals illustrated and described by Bruce (1990). In addition, the widest part of this species may vary between pereonite 4 and pereonite 5. This variation may also cause individual body shapes to appear dissimilar. The anterior margin of the cephalon of the syntype (MNHN–IU–2016–9885) appears to be rounded rather than subtruncate. The posterior margin of pleonite 5 can be roughly straight (AM G2181 from Bruce 1990), have a slight medial point, or be weakly concave (Bruce 1990, present study). Although Bruce (1990) described the uropods as being short, most measure more than half the length of the pleotelson.

*Size.* Ovigerous females 20.0–26.7 mm TL, 14.0–15.0 mm W. Other material: ovigerous females 22.0–67.0 mm TL (average 30.83 mm TL) (Bruce 1990).

####### Distribution.

Records listed from west to east. North Pacific Ocean: Bering Sea (Kensley 1976). South America: Punta Quillaipe (Menzies 1962) and Chile (Nierstrasz 1931); Uruguay (Mañé-Garzón 1979). South Atlantic Ocean: Saint Helena and Tristan da Cunha (Sivertsen and Holthuis 1980). South Africa: Table Bay (Barnard 1920); Cape of Good Hope (Milne Edwards 1840); Durban (Barnard 1955). India: Travancore (Pillai 1954). Southern Indian Ocean: Amsterdam Island (Kensley 1976). Australia: southern and south-eastern Australia (Schioedte and Meinert 1884, Hale 1926, Bruce 1990, Whitelegge 1901). Japan: Yokohama (Schioedte and Meinert 1884). New Zealand (Filhol 1885, Chilton 1909, Nierstrasz 1915, Hurley 1961, Bruce 1990).

####### Hosts.

*Elthusaraynaudii* has been recorded from various fish hosts of multiple orders and families. These hosts are: *Chelidonichthyskumu* (Cuvier, 1829) (see Avdeev 1978); *Chorisochismusdentex* (Pallas, 1769) (see Barnard 1920); *Cyttusaustralis* (Richardson, 1843) (see Avdeev 1978, 1984, Bruce 1990); *Cyttusnovaezelandiae* (Arthur, 1885) (see Avdeev 1978, 1984); *Cyttustraversi* Hutton, 1872, previously *Cyttoidopsmccullochi* (Whitley, 1947) (see Avdeev 1984, Bruce 1990); *Genypterusblacodes* (Bloch and Schneider, 1801) (see Hewitt and Hine 1972); *Gnathanacanthusgoetzeei* Bleeker, 1855 (see Bruce 1990); *Hyporhamphusintermedius* (Cantor, 1842) (see Powell 1959, Stephenson 1969); *Latrislineata* (Forster, 1801) (see Kensley 1976); *Meuscheniafreycineti* (Quoy and Gaimard, 1824) (see Bruce 1990); *Mustelusantarcticus* Günther, 1870 (see Hewitt and Hine 1972); *Nemadactylusmonodactylus* (Carmichael, 1819), previously *Acantholatrismonodactylus* (Carmichael, 1819) (see Sivertsen and Holthuis 1980); *Nematalosanasus* (Bloch, 1795) (see Ghani 2003); *Notacanthussexspinis* Richardson, 1846 (see Avdeev 1978, 1984); *Nototheniamicrolepidota* Hutton, 1875, previously *Nototheniacolbecki* (see Chilton 1909, Hewitt and Hine 1972, Avdeev 1978, 1984); *Notolabrustetricus* (Richardson, 1840), previously *Pseudolabrustetricus* (see Bruce 1990); *Paranototheniamagellanica* (Forster, 1801), previously *Nototheniamacrocephala* (see Avdeev 1978); *Ilishamelastoma* (Bloch and Schneider, 1801) previously *Pellonabrachysoma* (see Pillai 1954); *Pelotretisflavilatus* Waite, 1911 (see Chilton 1911); *Pseudophycisbachus* (Forster, 1801), previously *Physiculusbachus* (see Hewitt and Hine 1972); *Physiculus* sp. (see Bruce 1990); *Pseudophycisbarbata* Günther, 1863, previously *Physiculusbarbatus* (Günther, 1863) (see Bruce 1990); *Pseudolabrusmiles* (Schneider and Forster, 1801) (see Poore 1981, Bruce 1990); *Pseudophycisbachus* (Forster, 1801) (see Chilton 1911, Bruce 1990); *Rexeasolandri* (Cuvier, 1832) (see Bruce 1990); *Rhombosolea* sp. (see Hewitt and Hine 1972); *Sardinopssagax* (Jenyns, 1842), previously *Clupeaneopilchardus* Steindachner, 1879 (see Chilton 1911); *Scorpaenacardinalis* Solander and Richardson, 1842 (see Poore 1981); *Sebastescapensis* (Gmelin, 1789), previously *Sebastichthyscapensis* (Gmelin, 1789) (see Sivertsen and Holthuis 1980); *Stolephoruscommersonnii* Lacepède, 1803 (see Pillai 1954); *Thyrsitesatun* (Euphrasen, 1791) (see Sivertsen and Holthuis 1980); *Zenopsisnebulosa* (Temminck and Schlegel, 1845), previously *Zenopsisnebulosus* (see Bruce 1990); *Zeusfaber* Linnaeus, 1758 (see Hale 1926, Avdeev 1984). Unidentified by scientific names: banded perch (Serranidae), flathead (Platycephalidae) (see Bruce 1990).

####### Remarks.

*Elthusaraynaudii* can be distinguished by the cephalon having a narrowly truncate rostrum; pereonite 1 with anterior margin straight; pleonites subequal in shape and width; and broadly rounded uropod apices that extend to more than half the length of the pleotelson.

Originally described in 1840, from the Cape of Good Hope in South Africa, from an unknown host, *Elthusaraynaudii* has been recorded numerous times from a wide range of localities within the Indo-Pacific region. It is the only species of *Elthusa* that has been described from sub-Saharan Africa. It has been recorded from an unknown host from the Cape of Good Hope (see Milne Edwards 1840); from the rocksucker, *Chorisochismusdentex* (Pallas, 1769) near Cape Town (Table Bay) (see Barnard 1920); from a wrasse in Durban (see Barnard 1955); as well as from the striped trumpeter, *Latrislineata* (Forster, 1801) (see Kensley 1976).

*Elthusasigani* Bruce, 1990, which is only known from its type locality in Queensland, Australia, seems to be most similar to *E.raynaudii*. *Elthusasigani* can be distinguished from *E.raynaudii* by having an evenly concave pereonite 1 anterior margin; a flat, straight cephalon anterior margin; and coxae 7 that extend past the posterior margin of pereonite 7. In addition, *E.sigani* is a much smaller species in overall body length range (9.0–13.0 mm), compared to *E.raynaudii* (20.0–26.7 mm).

**Table 1.** Interspecific character states between *Elthusaraynaudii* (Milne Edwards, 1840), *Elthusaxena* sp. n., *Elthusaacutinasa* sp. n., and *Elthusarotunda* sp. n. from sub-Saharan African marine waters.

**Morphological feature**	***Elthusaraynaudii* (Milne Edwards, 1840)**	***Elthusaxena* sp. n.**	***Elthusaacutinasa* sp. n.**	***Elthusarotunda* sp. n.**
**Body shape**	Ovoid	Elongate ovoid	Elongate ovoid	Round
**Shape of cephalon and anterior margin**	Sub-truncate, blunt anterior margin	Sub-triangular, bluntly pointed anterior margin	Sub-triangular, pointed anterior margin	Sub-triangular, blunt anterior margin
**Pereonite 1 anterior margin**	Straight	Medially indented	Medial projection	Concave
**Coxae 7 posterior margin**	Not extending past posterior margin of pereonite 7	Not extending past posterior margin of pereonite 7	Extending past posterior margin of pereonite 7	Not extending past posterior margin of pereonite 7
**Pereopod 7 protrusions**	Absent	Absent	Absent	Present on merus and carpus
**Pleonite length**	Pleonites 1–5 sub-equal	Pleonite 5 longest and indented	Pleonite 1 longest	Pleonite 5 longest, medially convex
**Pleonite 1 width**	Narrower than other pleonites	As long as other pleonites	As wide as pleotelson	Narrower than other pleonites
**Pleonite 5 lateral margins**	Visible	Visible	Largely concealed by pleonite 4	Slightly concealed by pleonite 4
**Pleotelson shape**	Evenly rounded	Roughly quadrate and curved upwards	Rounded	Broadly rounded
**Pleopod 5 endopod**	Slightly smaller than exopod	Smaller than exopod (not dissected)	Half the size of exopod	Smaller than exopod (not dissected)
**Uropods**	Broadly rounded, more than half the length of pleotelson	Apices narrowly rounded, more than half the length of pleotelson	Short, pointed, less than half the length of pleotelson	Broadly rounded, half the length of pleotelson

###### Elthusa xena sp. n.

Taxon classificationAnimaliaIsopodaCymothoidae

http://zoobank.org/338A44A2-746F-4D9B-B890-5372D1E45B4C

**Figure 4.**
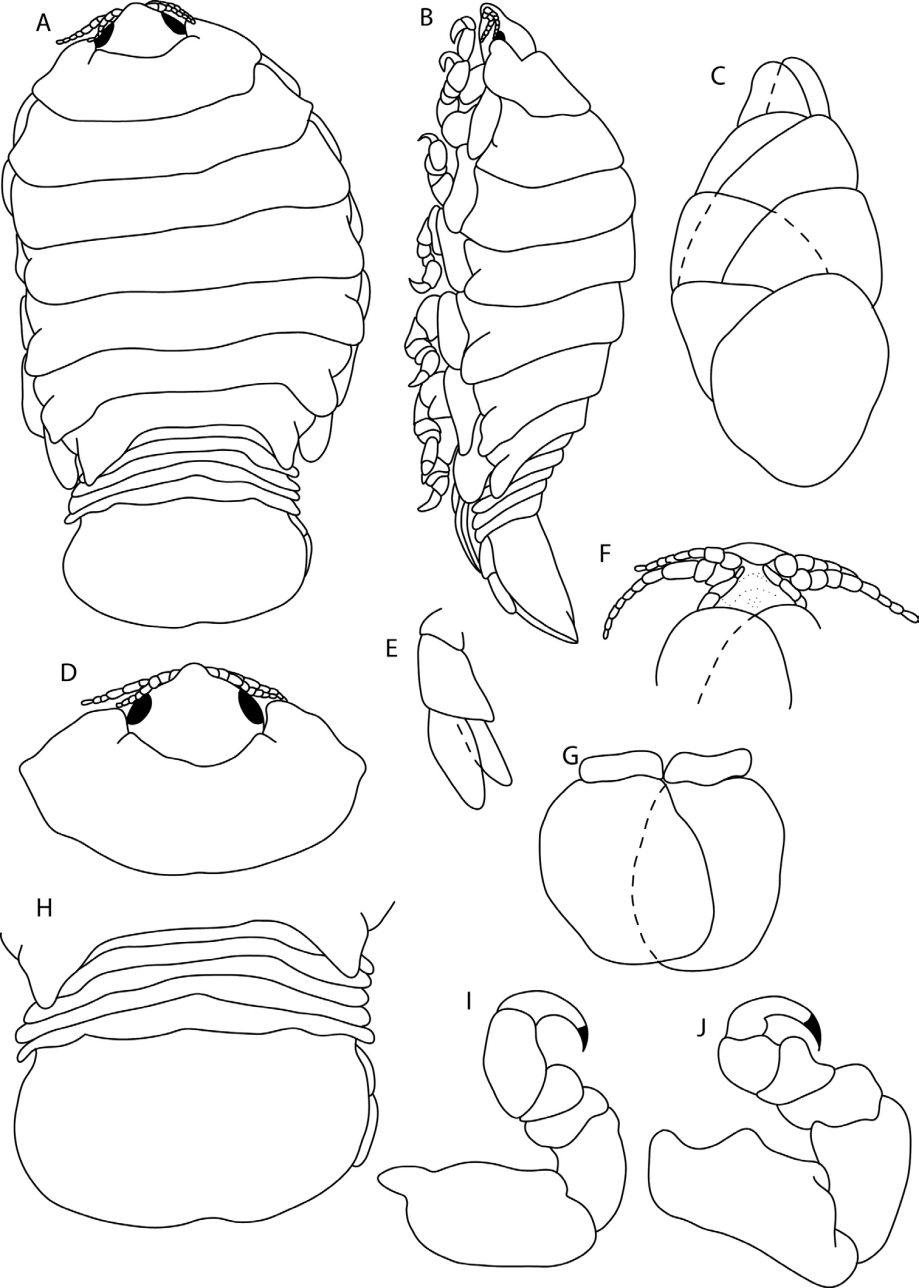
*Elthusaxena* sp. n. holotype ♀ (ovigerous, 34.0 mm TL, 17.0 mm W) (SAMC-A089958) from Alexander Bay, South Africa **A** dorsal body **B** lateral body **C** oostegites **D** dorsal view of cephalon and pereonite 1 **E** uropod **F** ventral cephalon **G** pleopod 1 **H** dorsal view of pleon **I** pereopod 1 **J** pereopod 7.

**Figure 5.**
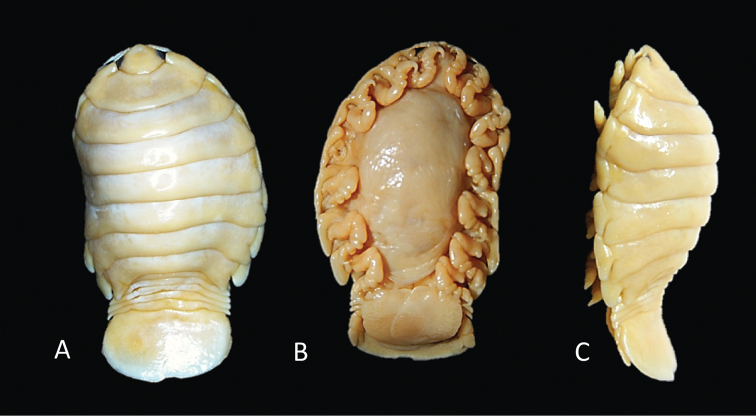
Photos of *Elthusaxena* sp. n. holotype ♀ (ovigerous, 34.0 mm TL, 17.0 mm W) (SAMC-A089958) from Alexander Bay, South Africa **A** dorsal view **B** ventral view **C** lateral view.

**Figure 6.**
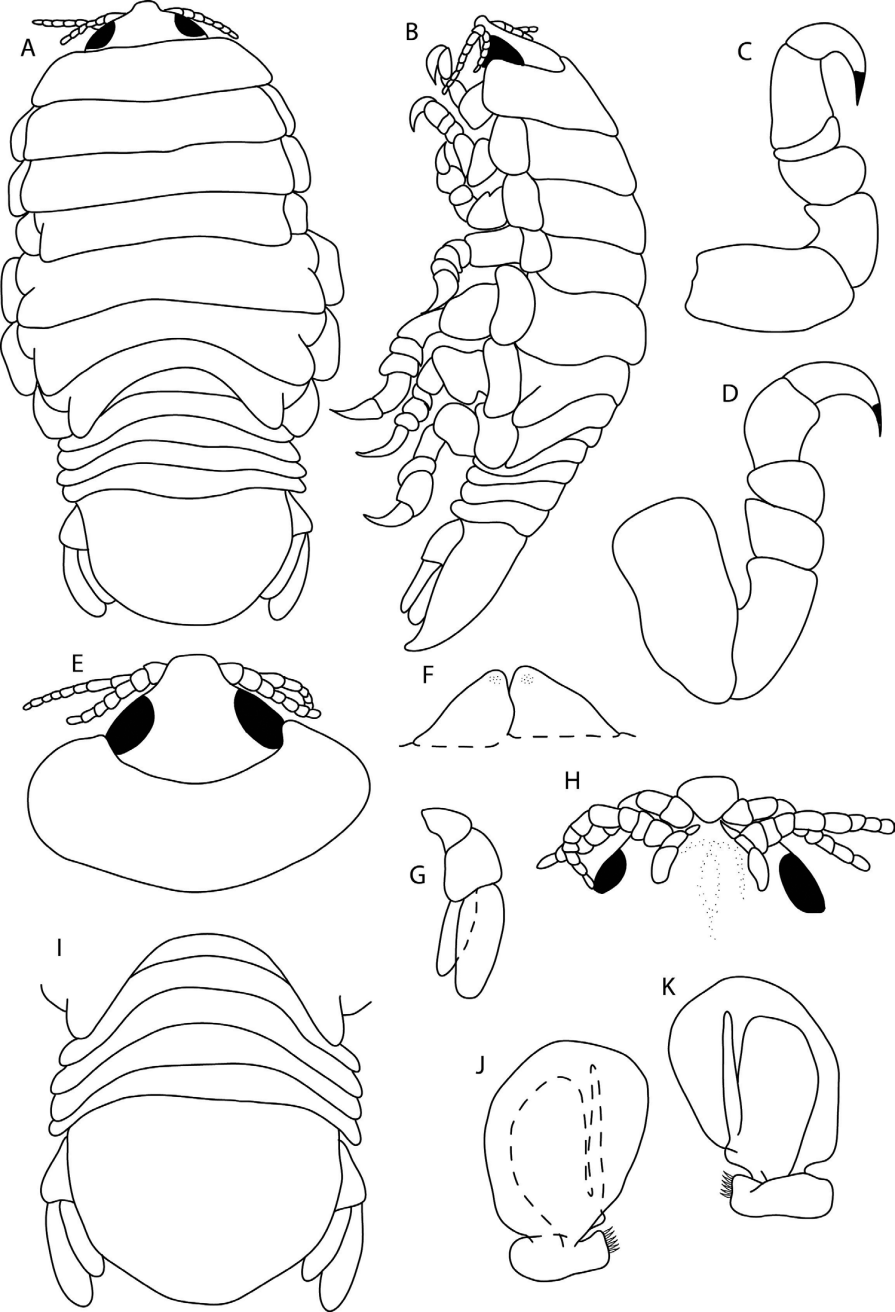
*Elthusaxena* sp. n. paratype ♂ (intermoult) (8 mm TL, 4 mm W) (SAMC-A089959) from Alexander Bay, South Africa **A** dorsal body **B** lateral body **C** pereopod 1 **D** pereopod 7 **E** dorsal view of cephalon **F** penes **G** uropod **H** ventral cephalon **I** dorsal view of pleon **J** ventral pleopod 2 **K** dorsal pleopod 2.

**Figure 7.**
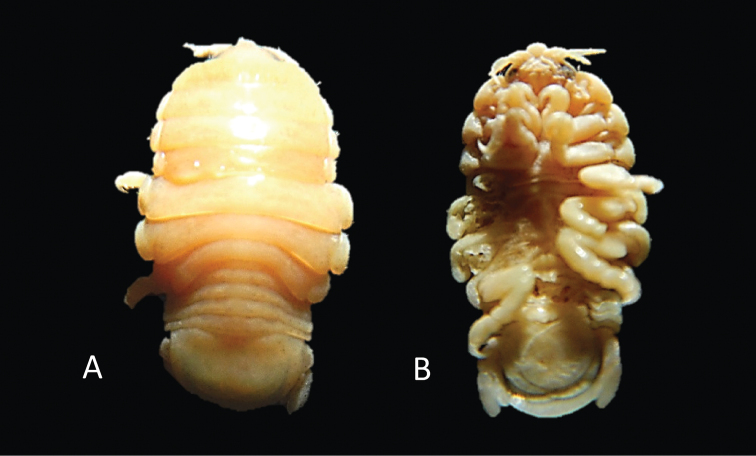
Photos of *Elthusaxena* sp. n. paratype ♂ (intermoult) (8.0 mm TL, 4.0 mm W) (SAMC-A089959) from Alexander Bay, South Africa **A** dorsal view **B** ventral view.

Figures 4 , 5 , 6 , 7 , Table 1

####### Material examined.

*Holotype*. SOUTH AFRICA • 1 ♀ (ovigerous, 34.0 mm TL, 17.0 mm W); Alexander Bay, mouth of the Orange River; 28°38'S, 16°27'E; July 1993; coll. J Laubscher; from the super klipfish, *Clinussuperciliosus* (Linnaeus, 1758); SAMC-A089958.

*Paratype*. SOUTH AFRICA • 1 ♂ (intermoult, 8.0 mm TL, 4.0 mm W); same data as holotype; SAMC-A089959.

####### Description

(ovigerous ♀). Figs 4–5. *Body* slightly twisted to the left, elongated ovoid, twice as long as greatest width; dorsal surfaces smooth and polished in appearance, widest at pereonite 5, most narrow at pereonite 1, pereonite lateral margins mostly rounded, medially indented. *Cephalon* 0.8 times longer than wide, visible from dorsal view, sub-triangular with blunt anterior point. *Frontal margin* thickened, ventrally folded. *Eyes* oval with distinct margins; one eye 0.1 times width of cephalon, 0.3 times length of cephalon. *Pereonite 1* smooth, anterior border slightly concave; anterolateral angle rounded, extending to the medial region of eyes. Posterior margins of pereonites smooth, slightly curved laterally. *Coxae* 2–3 narrow with posteroventral angles narrowly rounded; coxae 4–7 with rounded point, not extending past pereonite margin. Pereonites 2–5 subequal, pereonites 6 and 7 slightly narrower. *Pleon* 0.4 times as long as total body length, with pleonite 1 same width as other pleonites, lateral margins concealed by pereonite 7, slightly visible in dorsal view; pleonites posterior margin smooth, slightly curved laterally. *Pleonite 2* partially overlapped by pereonite 7; posterolateral angles of pleonite 2 rounded. *Pleonites 3–5* similar in form to pleonite 2; pleonite 5 longest, free, not overlapped by lateral margins of pleonite 4, with posterolateral angles narrowly rounded, posterior margin with 3 indentations. *Pleotelson* 0.6 times as long as anterior width, dorsal surface smooth; lateral margins convex; posterior margin evenly rounded.

*Antennula* shorter than antenna, consisting of eight articles; peduncle articles I and II distinct and articulated, extending to anterior of pereonite 1. *Antenna* consists of eleven articles, extending to past anterior margin of pereonite 1.

*Pereopod 1* basis 1.8 times as long as greatest width; ischium 0.7 times as long as basis; merus proximal margin without bulbous protrusion; carpus with rounded proximal margin; propodus 1.8 times as long as wide; dactylus slender, 0.8 times as long as propodus, 2.3 times as long as basal width. *Pereopods 2–3* similar to pereopod 1, all pereopods without robust or simple setae. *Pereopod 7* basis with carina, 1.5 times as long as greatest width; ischium without protrusions, 0.9 times as long as basis; merus proximal margin with slight bulbous protrusion, 0.6 times as long as wide, 0.3 times as long as ischium; carpus with bulbous protrusion, 0.9 times as long as wide, 0.5 times as long as ischium; propodus as long as wide, 0.4 times as long as ischium; dactylus slender, 1.9 times as long as propodus, 3.1 times as long as basal width.

*Pleopods* simple, exopod larger than endopod. *Pleopod 1* exopod 1.1 times as long as wide, lateral margin strongly convex, distally broadly rounded, mesial margin weakly convex; peduncle 2.8 times as wide as long.

*Uropod* more than half the length of pleotelson, peduncle 0.8 times longer than rami, peduncle lateral margin without setae; rami not extending beyond pleotelson, apices narrowly rounded. *Endopod* apically rounded, 2.5 times as long as greatest width, lateral margin weakly convex, mesial margin straight, terminating without setae. *Exopod* extending beyond end of endopod, twice as long as greatest width, apically rounded, lateral margin weakly convex, mesial margin straight, terminating without setae.

####### Description

(paratype intermoult ♂). Figs 6, 7. Male similar to female but smaller. Specimen mid-moult. *Body* rectangular, not twisted, twice as long as greatest width. Pereonite lateral margins mostly subparallel. *Cephalon* 0.7 times longer than wide. *Frontal margin* rounded to form blunt rostrum. *Eyes* oval with distinct margins; one eye 0.2 times width of cephalon; 0.5 times length of cephalon. *Pereonite 1* smooth, anterior border concave, extending past base of cephalon. Posterior margins of pereonites smooth and straight, except pereonite 4 and 5. *Coxae* 2–3 wide, with posteroventral angles rounded; coxae 4–7 rounded. Pereonites 6 and 7 narrower, becoming more progressively rounded posteriorly. *Pleon* 0.3 times as long as total body length, with pleonite 1 largely concealed by pereonite 7, slightly visible in dorsal view; pleonites 1–3 posterior margin posteriorly concave, smooth and slightly curved laterally. Pleonite 5 overlapped by lateral margins of pleonite 4, with posterolateral angles narrowly rounded, posterior margin straight. *Pleotelson* 0.8 times as long as anterior width, lateral margins straight or weakly convex, posterior margin broadly truncate.

*Antennula* shorter than antenna, consisting of eight articles. *Antenna* consists of ten articles, extending to middle of pereonite 1.

*Pereopod 1* basis twice as long as greatest width; ischium 0.6 times as long as basis; propodus 1.6 times as long as wide; dactylus 1.1 times as long as propodus, 3 times as long as basal width. *Pereopod 7* twice as long as greatest width; ischium 0.7 times as long as basis; merus proximal margin without bulbous protrusion, 0.7 times as long as wide, 0.4 times as long as ischium; carpus without bulbous protrusion, 0.7 times as long as wide, 0.4 times as long as ischium; propodus 1.3 times as long as wide, 0.6 as long as ischium; dactylus slender, 1.4 times as long as propodus, 2.7 times as long as basal width.

*Pleopod 1* exopod 1.2 times as long as wide, lateral margin weakly convex, distally broadly rounded, mesial margin straight; endopod 2.1 times as long as wide, lateral margin weakly convex, mesial margin straight, peduncle 2.2 times as wide as long. *Pleopod 2* appendix masculina with parallel margins, 1.1 times as long as endopod, distally narrowly rounded.

*Uropod* same length or slightly longer than the pleotelson, peduncle 0.4 times longer than rami, rami extending slightly beyond pleotelson, apices narrowly rounded. *Endopod* apically slightly pointed, 3 times as long as greatest width. *Exopod* 2.6 times as long as greatest width.

*Penes* medially adjacent; penial process 0.7 times as long as basal width.

####### Etymology.

The epithet is constructed in a possessive form of a personal name. This species is named after Xena, the warrior princess, in reference to the strong nature of the female cymothoid isopod.

####### Size.

Ovigerous female (34.0 mm TL, 17.0 mm W), male (8.0 mm TL, 4.0 mm W).

####### Distribution.

Currently only known from the mouth of the Orange River, Alexander Bay, South Africa (Atlantic Ocean).

####### Hosts.

*Clinussuperciliosus* (Linnaeus, 1758). This is the first record of a klipfish (of the genus *Clinus* Cuvier, 1816), and of the intertidal super klipfish, *Clinussupercilious*, as a fish host of a species of *Elthusa*. This host belongs to the fish order Perciformes, and is endemic to the Southeast Atlantic Ocean, from northern Namibia to the Kei River of South Africa (Smith and Heemstra 1986).

####### Remarks.

*Elthusaxena* sp. n. female can be identified by the elongate, ovoid body shape; coxae 7 that do not extend past the posterior margin of pereonite 7; a bluntly pointed anterior margin of the cephalon; evenly rounded, slightly concave anterior margin of pereonite 1; uropod rami with apices narrowly rounded and more than half the length of pleotelson; pleonite 5 posterior margin with indentations; and the pleotelson is short, roughly quadrate, with margins that curl upward.

Two other *Elthusa* species have been recorded from related perciform fish hosts from the family Clinidae Swainson, 1839 (blennies). *Elthusacalifornica* (Schioedte & Meinert, 1884) was noted from the striped kelpfish *Gibbonsiametzi* Hubbs, 1927; and *Elthusamenziesi* (Brusca, 1981) from both the spotted kelpfish *Gibbonsiaelegans* (Cooper, 1864) and the crevice kelpfish *Gibbonsiamontereyensis* Hubbs, 1927. However, this is the first record of *Elthusa* collected from a *Clinus* sp.

*Elthusaxena* sp. n. can be distinguished from *E.raynaudii* by having a bluntly pointed cephalon anterior margin, compared to the narrowly truncate margin of *E.raynaudii*. Other differences include the shape of the pleotelson (which is quadrate, wide and short for *E.xena* sp. n., and evenly rounded for *E.raynaudii*); pleonite 1 is the same length as the other pleonites in *Elthusaxena* sp. n. but narrower in *E.raynaudii*; and the uropod apices of *E.xena* sp. n. are narrowly rounded compared to the broadly rounded apices of *E.raynaudii* uropods. See Table 1 for further morphological variation and comparisons.

###### Elthusa acutinasa sp. n.

Taxon classificationAnimaliaIsopodaCymothoidae

http://zoobank.org/D5AFAEC4-F03D-400F-98A0-8D86631E495E

**Figure 8.**
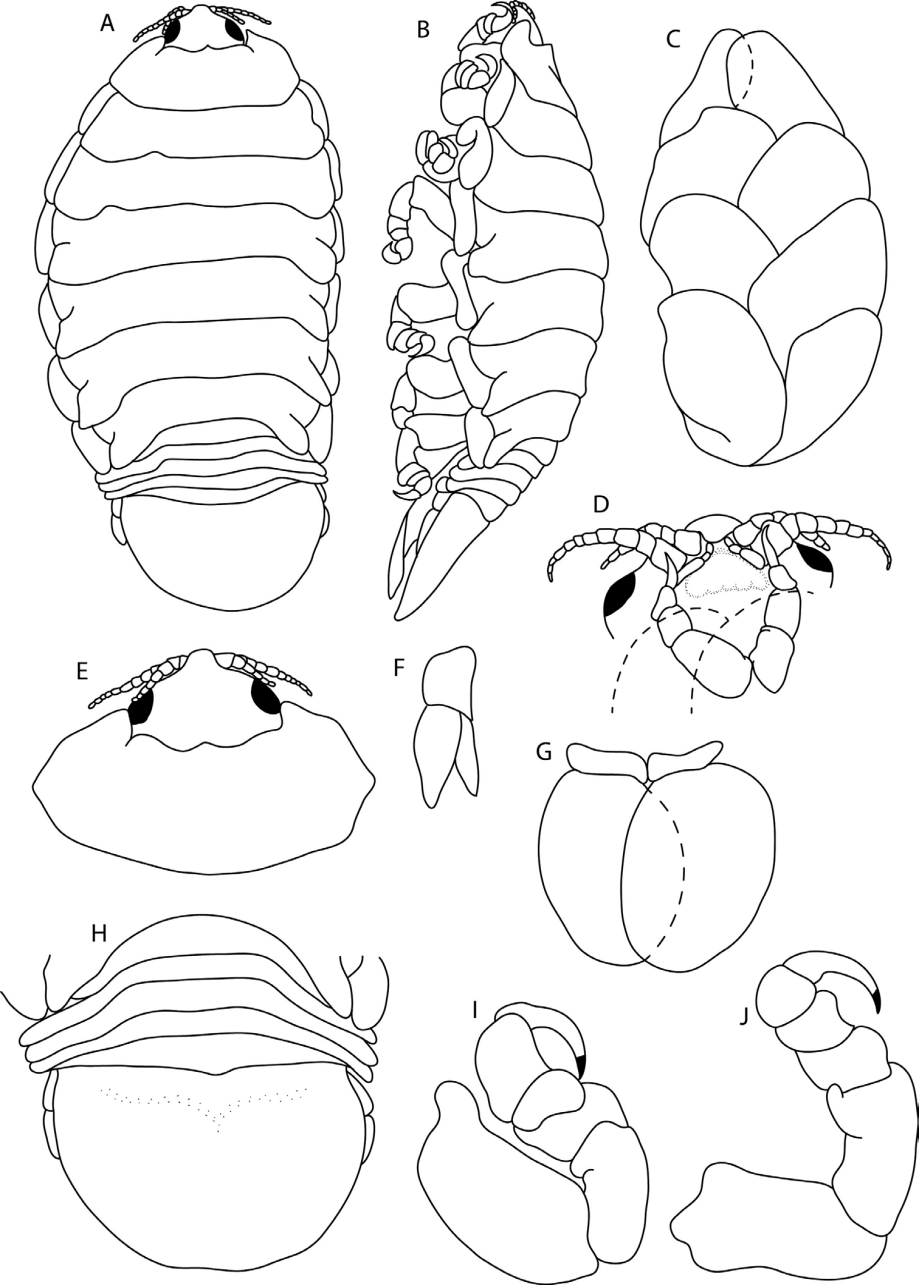
*Elthusaacutinasa* sp. n. holotype ♀ (ovigerous, 39.0 mm TL, 19.0 mm W) (SAMC-A089960) from Africana research vessel **A** dorsal body **B** lateral body **C** oostegites **D** ventral cephalon **E** dorsal view of cephalon and pereonite 1 **F** uropod **G** pleopod 1 **H** dorsal view of pleon **I** pereopod 1 **J** pereopod 7.

**Figure 9.**
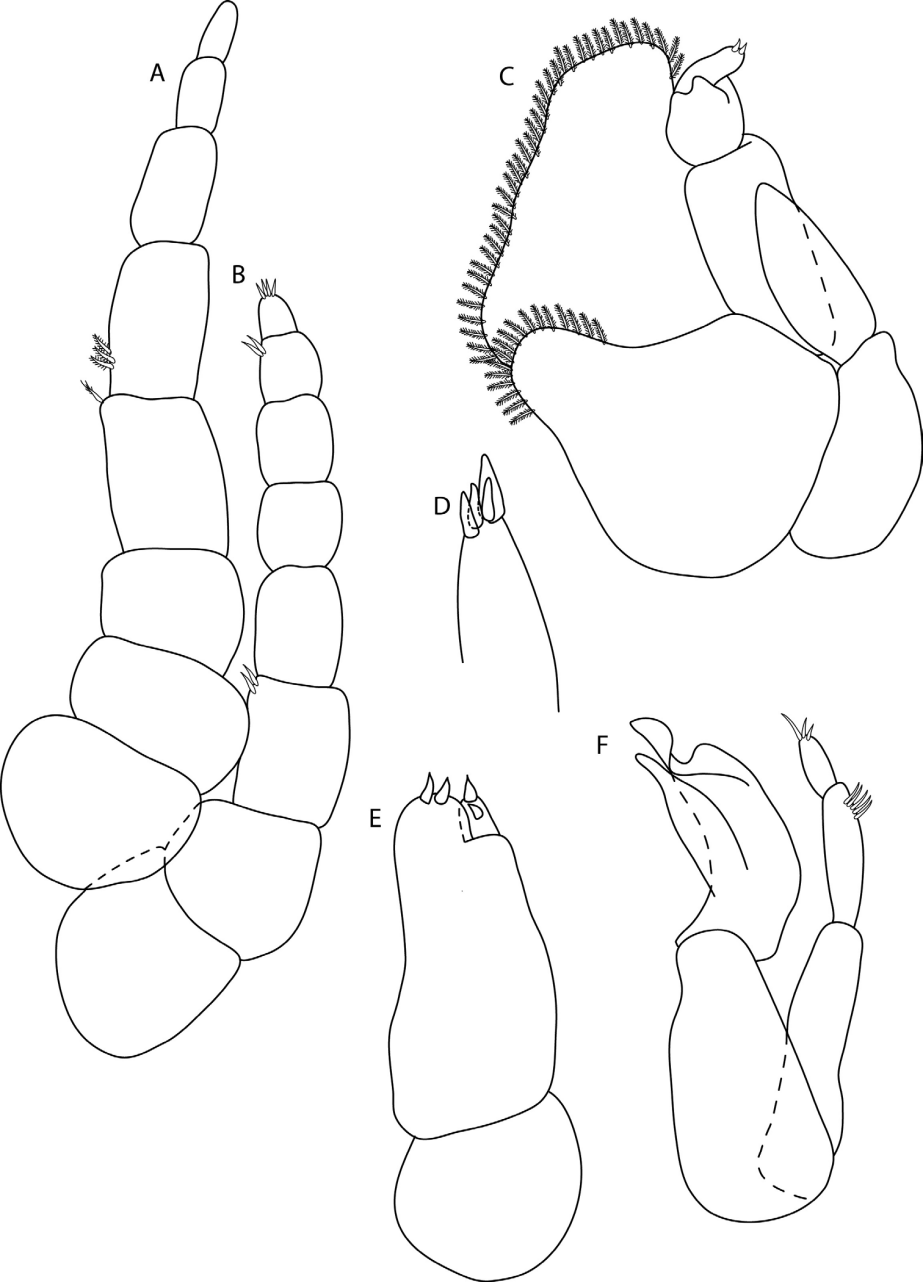
*Elthusaacutinasa* sp. n. paratype ♀ (ovigerous, 33.0 mm TL, 16.0 mm W) (SAMC-A089961) from Africana research vessel **A** antennula **B** antenna **C** maxilliped **D** tip of maxillula **E** maxilla **F** mandible.

**Figure 10.**
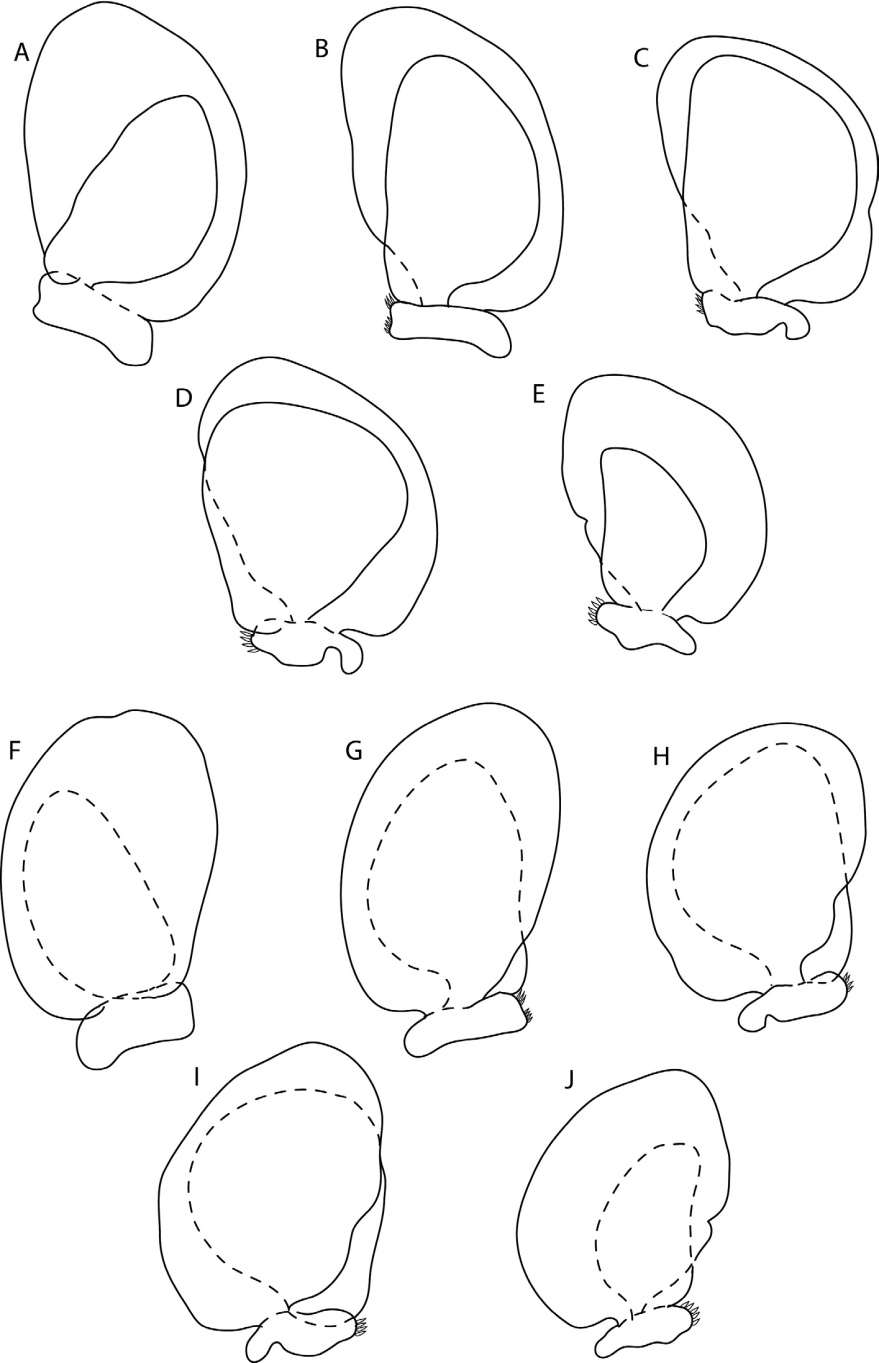
*Elthusaacutinasa* sp. n. paratype ♀ (ovigerous, 33.0 mm TL, 16.0 mm W) (SAMC-A089961) from Africana research vessel **A–E** dorsal view of pleopods 1–5 respectively **F–J** ventral view of pleopods 1–5 respectively.

**Figure 11.**
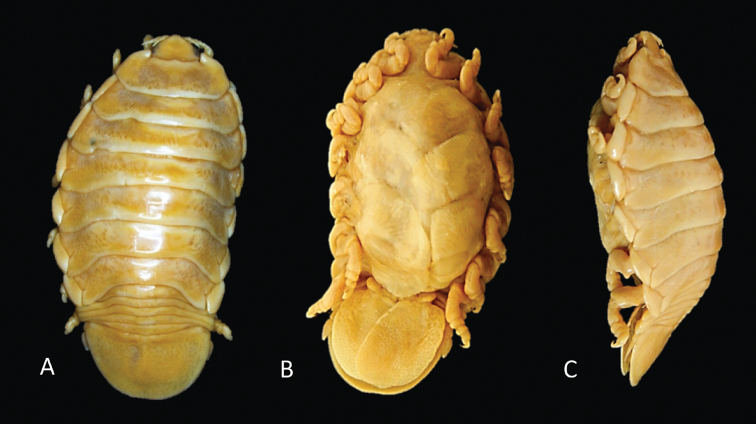
Photos of *Elthusaacutinasa* sp. n. holotype ♀ (ovigerous, 39.0 mm TL, 19.0 mm W) (SAMC-A089960) from Africana research vessel **A** dorsal view **B** ventral view **C** lateral view.

Figures 8 , 9 , 10 , 11 , Table 1

####### Material examined.

*Holotype*. SOUTH AFRICA • 1 ♀ (ovigerous, 39.0 mm TL, 19.0 mm W); Indian Ocean, south coast of South Africa, RV Africana (fish sorting table); 34°38'S, 25°38'E; April 2003; coll. Nico J Smit; SAMC-A089960.

*Paratypes*. SOUTH AFRICA • 3 ♀♀ (ovigerous, 28.0–30.0 mm TL, 15.0–17.0 mm W); same data as holotype; SAMC-A089961.

*Other material.* SOUTH AFRICA • 1 ♀ (ovigerous, 29.0 mm TL, 17.0 mm W); same data as holotype; dissected; in the collection of the authors at NWU • 4 ♀♀ (non-ovigerous, 19.0–24.0 mm TL, 10.0–14.0 mm W); same data as holotype; in the collection of the authors at NWU • 9 ♀♀ (three ovigerous, six non-ovigerous, 15.0–40.0 mm TL, 8.0–19.0 mm W); Indian Ocean, south coast of South Africa, RV Africana (fish sorting table); 30°29'S, 16°0'E; 213 m depth; January 1999; SAMC-A091307 • 1 ♀ (ovigerous, 40.0 mm TL, 19.0 mm W); same data as preceding; 30°25'S, 16°9'E; 259 m depth; SAMC-A091308 • 1 ♀ (ovigerous, 30.0 mm TL, 15.0 mm W); same data as preceding; 31°8'S, 15°20'E; 234 m depth; SAMC-A091309.

####### Description

(ovigerous ♀). Figs 8–11. *Body* slightly twisted to the right, elongated ovoid, 2.1 times as long as greatest width. Body dorsal surfaces smooth and polished in appearance, widest at pereonite 4, most narrow at pereonite 1, pereonite lateral margins mostly posteriorly ovate, medially indented. *Cephalon* 0.4 times longer than wide, visible from dorsal view, sub-triangular with narrowly rounded anterior point. *Frontal margin* thickened, ventrally folded. *Eyes* oval with distinct margins; one eye 0.2 times width of cephalon, 0.4 times length of cephalon. *Pereonite 1* smooth, anterior border with medially produced point, with two indentations; anterolateral angle rounded, extending to posterior margin of eyes. Posterior margins of pereonites smooth and slightly curved laterally. Coxae 2–3 wide; with posteroventral angles rounded; 4–7 with rounded point. Coxae 7 extending slightly past pereonite posterior margin. Pereonites 2–5 subequal, becoming more progressively rounded posteriorly. *Pleon* 0.4 times as long as total body length, with pleonite 1 longest, lateral margins concealed by pereonite 7, visible in dorsal view; pleonites posterior margin smooth and slightly curved laterally. *Pleonite 2* partially overlapped by pereonite 7; posterolateral angles of pleonite 2 rounded. Pleonites 3–5 similar in form to pleonite 2; pleonite 5 overlapped by lateral margins of pleonite 4, posterior margin straight, with slight medial point. *Pleotelson* 0.7 times as long as anterior width, dorsal surface smooth; lateral margins weakly convex; posterior margin rounded, with slight medial indent.

*Antennula* shorter than antenna, consisting of eight articles; antennula peduncle articles I and II distinct and articulated; article II 0.9 times as long as article 1; article III 1.4 times as long as wide, 0.5 times as long as combined lengths of articles I and II; antennula flagellum with five articles, extending to middle of eye, with tufts of setae on articles I–III and article VIII. *Antenna* consists of twelve articles. *Antenna* peduncle article III 1.3 times as long as article II; article IV 1.3 times as long as wide, 1.2 times as long as article III; article V 1.5 times as long as wide, 1.1 times as long as article IV. Antenna flagellum with six articles, terminal article terminating in 1–5 short simple setae, extending to past anterior margin of pereonite 1. *Mandible palp* article II with five distolateral setae, and article III with three simple setae. *Maxillula* simple with four terminal robust setae. *Maxilla* mesial lobe not fused to lateral lobe; lateral lobe without simple setae, two recurved robust setae; mesial lobe without simple setae, and two large recurved robust setae. *Maxilliped* consists of III articles, with lamellar oostegite lobe or second, smaller oostegite lobe on basal part of article, palp article II without simple setae, article III with three recurved robust setae. Oostegites margin covered in numerous plumose setae.

*Pereopod 1* basis 1.9 times as long as greatest width; ischium 0.7 times as long as basis; merus proximal margin with slight bulbous protrusion; carpus with rounded proximal margin; propodus 1.1 times as long as wide; dactylus slender, 1.3 times as long as propodus, 3 times as long as basal width. *Pereopod 3* similar to pereopod 2, all pereopods without robust or simple setae. *Pereopod 7* basis 1.9 times as long as greatest width; ischium with slight bulbous protrusion on distal margin, 0.9 times as long as basis; merus proximal margin with slight bulbous protrusion, 0.6 times as long as wide, 0.3 times as long as ischium; carpus with bulbous protrusion, 0.7 times as long as wide, 0.3 times as long as ischium; propodus 1 times as long as wide, 0.3 times as long as ischium; dactylus slender, 1.9 times as long as propodus, 3.3 times as long as basal width.

*Pleopods* simple; exopod larger than endopod, with 4–7 simple setae on peduncle of pleopods 2–5. *Pleopod 1* exopod 1.3 times as long as wide, lateral margin weakly convex, distally broadly rounded, mesial margin straight; peduncle 3 times as wide as long. *Endopod* 1.6 times as long as wide, lateral margin convex, distally narrowly rounded, mesial margin straight, peduncle 2.4 times as wide as long. *Pleopods 2–5* similar to pleopod 1, mesial margins becoming more strongly produced, peduncle lobes absent.

*Uropod* less than half the length of the pleotelson, peduncle 0.7 times longer than rami, peduncle lateral margin without setae, marginal setae absent, apices narrowly rounded. *Endopod* apically slightly pointed, 3.4 times as long as greatest width, lateral margin weakly convex, mesial margin straight, terminating without setae. *Exopod* extending to end of endopod, 2.3 times as long as greatest width, apically rounded, lateral margin distally convex, mesial margin straight, terminating without setae.

*Variations.* Intra-specific variation was observed among the examined specimens of *Elthusaacutinasa* sp. n. The size of the medial point formed at the anterior margin of pereonite 1 may vary. Some specimens portrayed an obvious, sharp medial point, while others only had a weak medial projection of the anterior margin of pereonite 1. Variation in the length of the uropods are slight, but one specimen had uropod rami extending to half the length of the pleotelson, while all the others specimens’ uropods were remarkably short. The overlapping of pleonite 5 lateral margins by pleonite 4 was consistent, except with one of the other examined paratype females, where pleonite 5 lateral margins were slightly visible. Some variation was also noted in the width of pleonite 1.

####### Etymology.

The epithet is a noun in the genitive singular. The species name *acutinasa* was derived by the son of one of us (NJS) from a combination of the two Latin words *acute* and *nasus*. The word acute translates to a feature that is pointy or ends with a sharp point; while *nasus* translates to nose. The combined word, *acutinasa*, therefore means pointy nose, and appropriately describes one of the characters of this species, which is its pointed anterior margin of the rostrum.

####### Size.

Ovigerous females (28.0–40.0 mm TL, 15.0–19.0 mm W), non-ovigerous females (19.0–24.0 mm TL, 10.0–14.0 mm W).

####### Distribution.

Known from the Indian Ocean, off the south coast of South Africa.

####### Hosts.

Not known (type material was collected from the fish sorting table following a trawl and not from a specific fish species).

####### Remarks.

*Elthusaacutinasa* sp. n. can be identified by its elongate, ovoid body shape; pointed anterior margin of the cephalon; anterior margin of pereonite 1 with short medial point; short, apically pointed uropod rami, which extend to less than half of the length of the pleotelson; coxae 7 that extends past the posterior margin of pereonite 7; pleonite 5 lateral margins that are largely concealed by pleonite 4; pleonite 5 posterior margin with a slight medial point; pleonite 1 the longest of the pleonites; and pleopod 5 endopod approximately half the size of the exopod.

Several characters differentiate between *E.acutinasa* sp. n. from *E.raynaudii* (see Table 1). *Elthusaacutinasa* sp. n. has a prominent, pointed cephalon anterior margin with a medially pointed pereonite 1 anterior margin compared to the straight anterior margin of *E.raynaudii* cephalon and pereonite 1. Pleon differences include the longer pleotelson of *E.acutinasa* sp. n. with pleonite 1 widest and pleonite 5 lateral margins concealed by those of pleonite 4 (not seen in *E.raynaudii*). *Elthusaacutinasa* sp. n. also has short uropods that do not extend to the half of the pleotelson length, whereas those of *E.raynaudii* reach to, or extend past, the half of the pleotelson length.

*Elthusaacutinasa* sp. n. can also be distinguished from *E.xena* sp. n. by its short uropods and coxae 7 that extend past the posterior margin of pereonite 7. Further differences are found within pleon morphology, where *E.acutinasa* sp. n. pleonite 5 lateral margins are largely concealed by pleonite 4, whereas those of *E.xena* sp. n. are visible. Pleonite 1 in *E.xena* sp. n. is as wide as the other pleonites, whereas pleonite 1 in *E.acutinasa* sp. n. is narrower than the other pleonites. The pleotelson shape of *E.acutinasa* sp. n. is evenly rounded, compared to the roughly quadrate pleotelson of *E.xena* sp. n. (see Table 1).

###### Elthusa rotunda sp. n.

Taxon classificationAnimaliaIsopodaCymothoidae

http://zoobank.org/138FBF0D-2E4B-4561-86C8-F209B78A33E0

**Figure 12.**
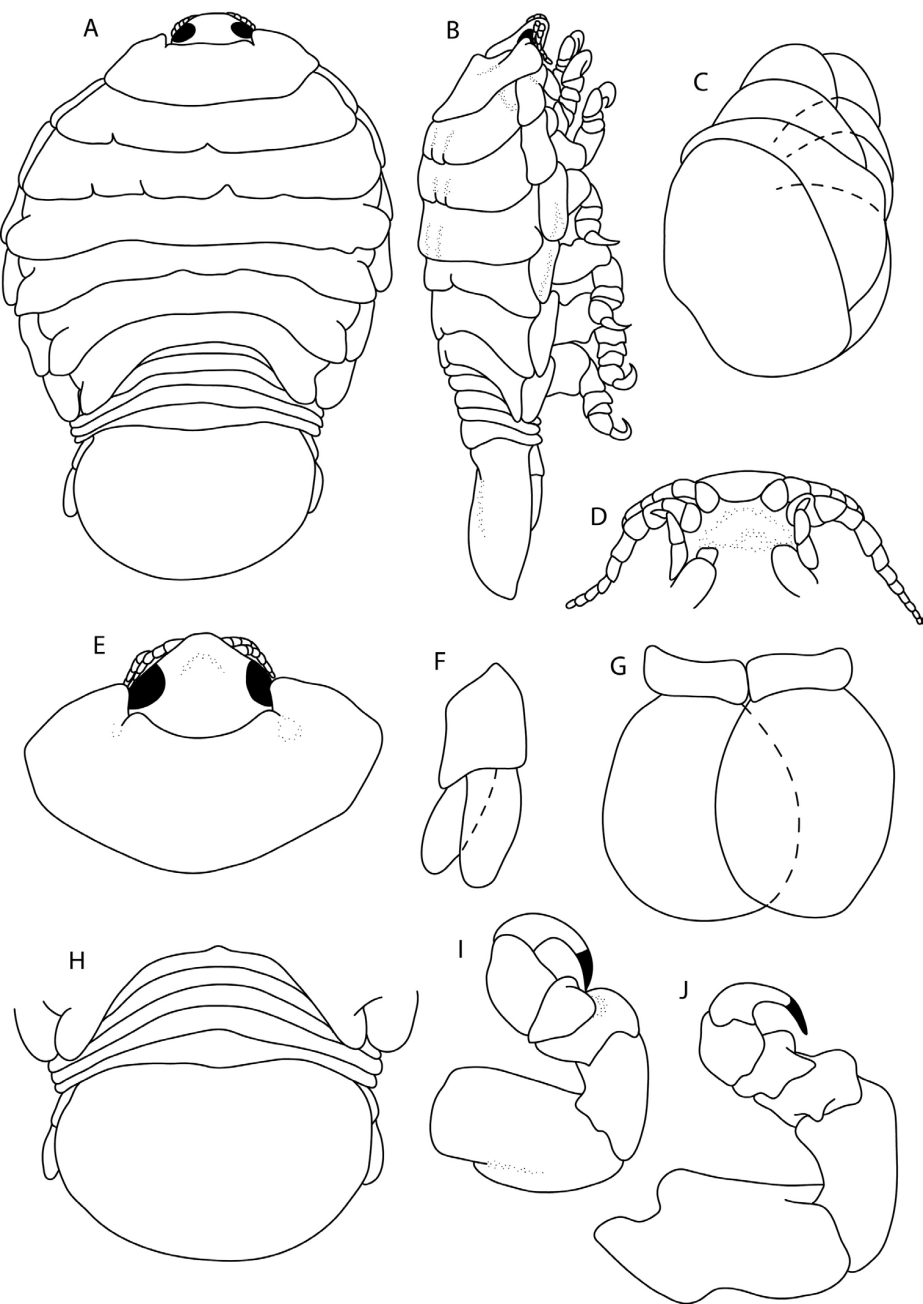
*Elthusarotunda* sp. n. holotype ♀ (ovigerous, 28 mm TL, 19 mm W) (SAMC-A11001) from Sea Point, South Africa **A** dorsal body **B** lateral body **C** oostegites **D** ventral cephalon **E** dorsal view of cephalon and pereonite 1 **F** uropod **G** pleopod 1 **H** dorsal view of pleon **I** pereopod 1 **J** pereopod 7.

**Figure 13.**
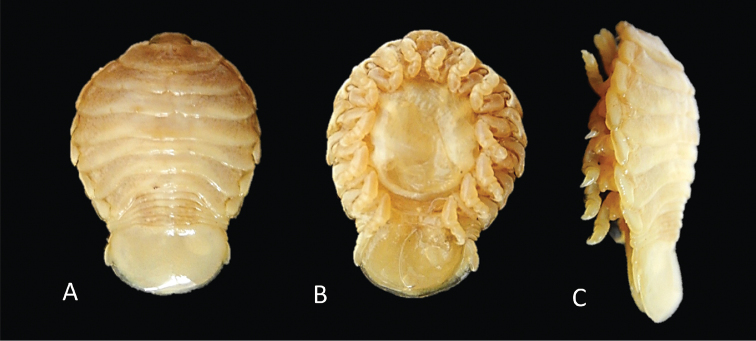
Photos of *Elthusarotunda* sp. n. holotype ♀ (ovigerous, 28 mm TL, 19 mm W) (SAMC-A11001) from Sea Point, South Africa **A** dorsal view **B** ventral view **C** lateral view.

Figures 12 , 13 , Table 1

####### Material examined.

*Holotype*. SOUTH AFRICA • 1 ♀ (ovigerous, 29.0 mm TL; 20.0 mm W); Cape Town, Sea Point; 33°55'S, 18°23'E; January 1960; coll. G Branch; SAMC A11001.

####### Description

(ovigerous ♀). Figs 12–13. *Body* round, not twisted, 1.4 times as long as greatest width; dorsal surfaces smooth and polished in appearance, widest at pereonite 4, most narrow at pereonite 1; pereonite lateral margins mostly posteriorly ovate, medially indented. *Cephalon* 0.4 times longer than wide, visible from dorsal view, sub-triangular with blunt anterior point. *Frontal margin* thickened, ventrally folded. *Eyes* oval with distinct margins; one eye 0.2 times width of cephalon; 0.5 times length of cephalon. *Pereonite 1* smooth, anterior border evenly concave; anterolateral angles rounded, extending to the medial region of eyes. Posterior margins of pereonites smooth, slightly curved laterally, posterior margins of pereonites 2–3 uneven. Coxae 2–3 wide; with posteroventral angles rounded; coxae 4–7 with rounded point, not extending past pereonite posterior margin. Pereonites becoming more progressively rounded posteriorly; pereonite 5 most narrow. *Pleon* 0.4 times as long as total body length; pleonite 1 largely concealed by pereonite 7, slightly visible in dorsal view; pleonites posterior margin slightly concave, smooth, slightly curved laterally. Pleonite 2 lateral margins overlapped by pereonite 7. Pleonites 3–4 similar in form to pleonite 2; pleonite 5 longest, overlapped by lateral margins of pleonite 4, posterior margin medially convex. *Pleotelson* broadly rounded, 0.7 times as long as anterior width, dorsal surface smooth; lateral margins convex; posterior margin evenly rounded.

*Antennula* shorter than antenna, consisting of eight articles; peduncle articles I and II distinct and articulated; extending to middle of eye. *Antenna* consists of ten articles, extending to past anterior margin of pereonite 1.

*Pereopod 1* basis 1.7 times as long as greatest width; ischium 0.7 times as long as basis; merus proximal margin without bulbous protrusion; propodus 1.4 times as long as wide; dactylus slender, 1.3 times as long as propodus, 2.9 times as long as basal width. All pereopods without robust or simple setae. *Pereopod 7* basis with carina, 2.1 times as long as greatest width; ischium with slight bulbous protrusion, 0.8 times as long as basis; merus proximal margin with bulbous protrusion, 0.6 times as long as wide, 0.3 times as long as ischium; carpus with bulbous protrusion, 0.7 times as long as wide, 0.3 times as long as ischium; propodus 1.2 times as long as wide, 0.9 times as long as ischium; dactylus slender, 1.7 times as long as propodus, 2.5 times as long as basal width.

*Pleopods* simple, exopod larger than endopod. *Pleopod 1* exopod 1.3 times as long as wide, lateral margin weakly convex, distally broadly rounded, mesial margin weakly convex; peduncle 2.5 times as wide as long.

*Uropod* half the length of pleotelson, peduncle 0.9 times longer than rami, peduncle lateral margin without setae; rami not extending beyond pleotelson, marginal setae absent, apices broadly rounded. *Endopod* apically rounded, 2.6 times as long as greatest width, lateral margin weakly convex, mesial margin weakly convex. *Exopod* extending to end of endopod, 2.2 times as long as greatest width, apically rounded, lateral margin weakly convex, mesial margin straight.

####### Size.

Ovigerous female (29.0 mm TL, 20.0 mm W).

####### Etymology.

The epithet is a noun in the nominative singular. It is named after its most distinct, defining character, which is the rounded shape of the body. The Latin word for round is *rotundus*.

####### Distribution.

Currently only known from Sea Point, Cape Town, South Africa.

####### Hosts.

Not known.

####### Remarks.

The diagnostic characters of *E.rotunda* sp. n. include its circular body shape; a sub-triangular cephalon with blunt anterior margin; pereopod 7 merus and carpus with protrusions on the proximal and lateral margins; pereonite 7 lateral margins that extend to pleonite 4; pleonite 5 longest and medially convex; a broadly rounded pleotelson posterior margin; and uropod rami that are sub-equal in length to the peduncle.

When comparing *E.rotunda* sp. n. to the rest of the identified *Elthusa* species, its closest resemblance is to that of *E.raynaudii*. This is especially in regards to the shape of the uropods, pleon, and cephalon anterior margin. It can be distinguished from *E.raynaudii* in having a more rounded body shape compared to the ovoid body shape of *E.raynaudii*; triangular cephalon as opposed to the narrowly truncate cephalon of *E.raynaudii*; the broadly rounded pereonite 1 anterolateral margins of *E.rotunda* sp. n. compared to the narrowly rounded to pointed anterolateral margins of *E.raynaudii* pereonite 1; as well as the uropod rami and peduncles that are subequal in length, as opposed to the longer rami of *E.raynaudii* (see Table 1).

*Elthusarotunda* sp. n. can be distinguished from *E.xena* sp. n. by the cephalon anterior margin which is more pointed in *E.xena* sp. n. and more rounded in *E.rotunda* sp. n.; broadly rounded uropod apices compared to the narrowly rounded ones from *E.xena* sp. n.; the shape of the pleotelson, which is broadly rounded for *E.rotunda* sp. n. and roughly quadrate for *E.xena* sp. n.; as well as the prominent presence of pereopod 7 protrusions on the merus and carpus of *E.rotunda* sp. n., that are less bulbous on *E.xena* sp. n.

The main differentiating characters between *E.rotunda* sp. n. and *E.acutinasa* sp. n. include the shape of the cephalon anterior margin (bluntly rounded versus produced point); and the uropod morphology, with *E.rotunda* sp. n. having broadly rounded, longer uropodal rami in comparison to the short, pointed uropodal rami of *E.acutinasa* sp. n. *Elthusarotunda* sp. n. pleonite 5 is the longest, whereas *E.acutinasa* sp. n. pleonite 1 is the longest; the presence of pereopod 7 protrusions on *E.rotunda* sp. n. is more prominent and bulbous that those of *E.acutinasa* sp. n. pereopod 7 (see Table 1).

## Conclusions

From previous collections across South Africa, four *Elthusa* species were recognised. *Elthusaraynaudii*, the only known *Elthusa* species from South Africa, was identified along with three new species from this genus. These new species, *E.xena* sp. n., *E.acutinasa* sp. n., and *E.rotunda* sp. n., more than double the known records of *Elthusa* from this region. Descriptions were provided for the three new *Elthusa* species along with an identification key with diagnostic characters to distinguish between the sub-Saharan *Elthusa* species (Table 1). A summative table was provided with currently known information on all species from the genus *Elthusa*, including host and location records of each (Table 2).

**Table 2.** Summary of the hosts, distribution, and attachment sites of all 33 species from the genus *Elthusa* Schioedte & Meinert, 1884, as well as the references for each record.

Species	Distribution	Hosts	References
*Elthusaalvaradoensis* Rocha-Ramírez, Chávez-López & Bruce, 2005	**TLoc**: Alvarado, Veracruz, Mexico.	**TH**: *Synodusfoetens* (Linnaeus, 1766)	Rocha-Ramírez et al. (2005)
*Elthusaarnoglossi* Trilles & Justine, 2006	**TLoc**: Chesterfield Islands, New Caledonia.	**TH**: *Arnoglossus* sp.	Trilles and Justine (2006)
*Elthusaatlantniroi* (Kononenko, 1988)	**TLoc**: Bay of Biscay, northeast Atlantic Ocean	**TH**: *Cepolamacrophthalma* (Linnaeus, 1758)	Kononenko (1988)
*Elthusacalifornica* (Schioedte & Meinert, 1884) **Syn**: *Livonecacalifornica* Schioedte & Meinert, 1884	**TLoc**: California, near San Francisco	**TH**: *Holconoti* sp.	Schioedte and Meinert (1884); Keys (1928); Hatch (1947); Olson (1972); Iverson (1974); Miller (1975); Waugh et al. (1989); Bennett (1993); Brusca (1981); Brusca et al. (2001); Gamble et al. (2013)
	**OL**: Pacific coast from Alaska to Peru; Canada; USA; Mexico	**OH**: Species from the families Atherinidae; Aulorhynchidae; Clinidae; Clupeidae; Cottidae; Embiotocidae; Fundulidae; Gasterosteidae; Gobiidae; Hexagrammidae; Moronidae; Mugilidae; Pholidae; Osmeridae; Paralichthyidae; Pholidae; Pleuronectidae; Sebastidae	
*Elthusacaudata* (Schioedte & Meinert, 1884) **Syn**: *Livonecacaudata* Schioedte & Meinert, 1884	**TLoc**: Laponica islands, Japan	**TH**: Unknown	Schioedte and Meinert (1884); Avdeev (1978)
	**OL**: New Zealand	**Other hosts**: *Genypterusblacodes* (Forster, 1801)	
*Elthusaemarginata* (Bleeker, 1857) **Syn**: *Livonecaemarginata* Bleeker, 1857	**TLoc**: Java, Indonesia	**TH**: Unknown	Bleeker (1857); Miers (1881); Schioedte and Meinert (1884); Nierstrasz (1915); Trilles and Randall (2011)
	**OL**: East India; Malaysia; Indonesia	**OH**: Species from the family Mullidae	
*Elthusaepinepheli* Trilles & Justine, 2010	**TLoc**: Off Nouméa, New Caledonia	**TH**: *Epinephelushowlandi* (Günther, 1873)	Trilles and Justine (2010)
*Elthusafoveolata* (Hansen, 1897) **Syn**: *Ironafoveolata* Hansen, 1897	**TLoc**: Sri Lanka	**TH**: Unknown	Hansen (1897)
*Elthusafrontalis* (Richardson, 1910) **Syn**: *Livonecafrontalis* Richardson, 1910	**TLoc**: Sablayan, Philippines	**TH**: *Balistes* sp.	Richardson (1910)
*Elthusamenziesi* (Brusca, 1981) **Syn**: *Lironecamenziesi* Brusca, 1981	**TLoc**: San Quintin Bays, Baja California, Mexico	**TH**: *Clinocottusanalis* (Girard, 1858)	Brusca (1981); Ruiz-Campos (1986); Wetzer et al. (1991); Espinosa-Pérez and Hendrickx (2001)
	**OL**: Mexico and Western Baja California	**OH**: Species from the families of Atherinidae; Blenniidae; Clinidae; Cottidae; Gobiesocidae; Kyphosidae; Labrisomidae; Lessoniaceae	
*Elthusamethepia* (Schioedte & Meinert, 1884)	**TLoc**: Rio de Janeiro, Brazil	**TH**: *Achirus* sp.	Schioedte and Meinert (1884)
*Elthusamoritakii* Saito & Yamauchi, 2016	**TLoc**: Honshu and east China Sea coast of Kyushu, Japan	**TH**: *Ereuniasgrallator* Jordan & Snyder, 1901	Saito and Yamauchi (2016)
*Elthusamyripristae* Bruce, 1990	**TLoc**: Escape Reef, outer Barrier Reef, Australia	**TH**: *Myripristisviolaceus* Bleeker, 1851	Bruce (1990)
*Elthusananoides* (Stebbing, 1905) **Syn**: *Ironananoides* Stebbing, 1905	**TLoc**: Galle, Sri Lanka (old Ceylon)	**TH**: Unknown	Stebbing (1905); Monod (1923); Trilles (1976)
	**OL**: Gulf of Suez, Red Sea	**OH**: Species from the families Holothuriidae; Leiognathidae; Molidae; Plotosidae; Scorpaenidae; Sparidae	
*Elthusaneocytta* (Avdeev, 1975) **Syn**: *Lironecaneocytta* Avdeev, 1975	**TLoc**: New Zealand	**TH**: *Neocyttusrhomboidalis* Gilchrist, 1906	Avdeev (1975, 1984); Stephenson (1987); Bruce (1990)
	**OL**: Tasmania and south-east New Zealand	**OH**: species from the families Cyttidae; Oreosomatidae; Scombridae; Zeidae	
*Elthusanierstraszi* Hadfield, Bruce & Smit, 2016 **Syn**: *Lironecaparva* Nierstrasz, 1915.	**TLoc**: Kisar Island, Moluccas, Indonesia	**TH**: *Ereuniasgrallator* Jordan & Snyder, 1901	Nierstrasz (1915); Avdeev (1984); Hadfield et al. (2016a)
*Elthusaochotensis* (Kussakin, 1979) **Syn**: *Lironecaochotensis* Kussakin, 1979	**TLoc**: Sea of Ochosk (near the city of Ayan), western Pacific Ocean	**TH**: Unknown	Kussakin (1979)
*Elthusaparabothi* Trilles & Justine, 2004	**TLoc**: New Caledonia, off Coëtlogon Bank	**TH**: *Parabothuskiensis* (Tanaka, 1918)	Trilles and Justine (2004)
*Elthusaparva* (Richardson, 1910) **Syn**: *Ceratothoaparva* (Richardson, 1910)	**TLoc**: Opol, Mindanao, Philippines	**TH**: Unknown	Richardson (1910); Hadfield et al. (2016b)
*Elthusaphilippinensis* (Richardson, 1910) **Syn**: *Livonecaphilippinensis* Richardson, 1910	**TLoc**: Jolo Light, Philippines	**TH**: Unknown	Richardson (1910)
*Elthusapoutassouiensis* (Penso, 1939) **Syn**: *Ceratothoapoutassouiensis* (Penso, 1939)	**TLoc**: Babakale Port, Aegean Sea Coasts, Turkey	**TH**: *Micromesistiuspoutassou* (Risso, 1827)	Brian (1939); Penso (1939); Öktener et al. (2018b)
	**OL**: Genova Gulf, Italy		
*Elthusapropinqua* (Richardson, 1904) **Syn**: *Livonecapropinqua* Richardson, 1904	**TLoc**: Port Heda, Japan	**TH**: Unknown	Richardson (1904, 1910); Barnard (1936); Bruce (1990)
	**OL**: Arabian Sea; Laccadive Islands; India; Maldives; Myanmar; Japan; Philippines; Australia	**OH**: “*chalinura*”; “*a macrurid*”, “*Macrurus*”; Ventrifossacf.nigrodorsalis	
*Elthusaraynaudii* (Milne Edwards, 1840) **Syn**: *Livonecaraynaudii* Milne Edwards, 1840	**TLoc**: Cape of Good Hope, South Africa	**TH**: Unknown	See in text.
	**OL**: See text	**OH**: See text	
*Elthusasacciger* (Richardson, 1909) **Syn**: *Livonecasacciger* Richardson, 1909	**TLoc**: Bungo Channel; Japan	**TH**: *Synaphobranchus* sp.	Avdeev (1984); Bruce (1990); Hata et al. (2017); Richardson (1909); Shiino (1951); Yamauchi (2009)
	**OL**: North-western Pacific; Australia; Japan and Pacific coast	**OH**: Species from the families Synaphobranchidae; Sebastidae	
*Elthusasamariscii* (Shiino, 1951) **Syn**: *Lironecasamariscii* Shiino, 1951	**TLoc**: Japan	**TH**: *Samariscusjaponicus* Kamohara, 1936	Shiino (1951); Biju Kumar and Bruce (1997)
	**OL**: Kerala coast, India	**Other hosts**: *Samariscristatus* Gray, 1831	
*Elthusasamoensis* (Schioedte & Meinert, 1884) **Syn**: *Livonecasamoensis* Schioedte & Meinert, 1884	**TLoc**: Samoa Islands (Samoenses islands)	**TH**: Unknown	Schioedte and Meinert (1884)
*Elthusasigani* Bruce, 1990	**TLoc**: North Stradbroke Island, Moreton Bay, southeastern Queensland, Australia	**TH**: *Siganusspinus* (Linnaeus, 1758)	Bruce (1990)
*Elthusasinuata* (Koelbel, 1879) **Syn**: *Livonecasinuata* Koelbel, 1879	**TLoc**: Mediterranean coast	**TH**: *Cepolamacrophthalma* (Linnaeus, 1758)	Koelbel (1879); Schioedte and Meinert (1884); Carus (1885); Gourret (1891); Gerstaecker (1901); Galati-Mosella (1920); Brian (1921); Monod (1924); Trilles (1968, 1977, 2008); Trilles and Raibaut (1973); Dollfus and Trilles (1976); Rokicki (1984, 1985); Trilles et al. (1989); Bello and Mariniello (1998); Trilles and Öktener (2004); Öktener et al. (2009, 2018a)
	**OL**: North-West Africa; United Kingdom; Mediterranean; Adriatic Sea; Spain; France; Algeria; Tunisia; Italy; Yugoslavia; Montenegro; Turkey	**OH**: Species from the families Argentinidae; Bramidae; Cepolidae; Gobiidae; Loliginidae; Pleuronectidae; Rajidae; Sepiolidae; Sparidae; Trichiuridae	
*Elthusasplendida* (Sadowsky & Moreira, 1981) **Syn**: *Lironecasplendida* Sadowsky & Moreira, 1981	**TLoc**: South Western Atlantic Ocean	**TH**: *Squaluscubensis* Howell Rivero, 1936	Sadowsky and Moreira (1981)
*Elthusatropicalis* (Menzies & Kruczynski, 1983) **Syn**: *Lironecatropicalis* Menzies & Kruczynski, 1983	**TLoc**: off Egmont Key, Florida, USA	**TH**: *Ogcocephalusparvus* Longley & Hildebrand, 1940	Menzies and Kruczynski (1983)
*Elthusaturgidula* (Hale, 1926) **Syn**: *Livonecaturgidula* Hale, 1926	**TLoc**: Western Australia	**TH**: Unknown	Hale (1926); Bruce (1990)
	**OL**: One Tree Island, Great Barrier Reef	**OH**: Species from the families Scaridae; Scaridae	
*Elthusavulgaris* (Simpson, 1857) **Syn**: *Livonecavulgaris* Stimpson, 1857	**TLoc**: San Francisco Bay; Tomales Bay; Monterey	**TH**: Unknown	Stimpson (1857); Richardson (1904); Turner et al. (1969); Hobson (1971); Brusca (1978, 1981); Bennett (1993); Espinosa-Pérez and Hendrickx (2001); Gamble et al. (2013)
	**OL**: Pacific Ocean including the western coast of USA, Mexico and Colombia	**OH**: Species from the families Carangidae; Chaenopsidae; Cottidae; Cynoglossidae; Embiotocidae;Engraulidae; Gobiidae; Hexagrammidae; Moronidae; Paralichthyidae; Pleuronectidae; Scorpaenidae; Sebastidae; Serranidae; Synodontidae. Also “rock cod”, “flounder”, “lingcod”	
*Elthusawinstoni* Hadfield, Tuttle & Smit, 2017	**TLoc**: Hawaii	**TH**: *Ctenochaetusstrigosus* (Bennett, 1828); *Acanthurusnigroris* Valenciennes, 1835	Hadfield et al. (2017)

## Supplementary Material

XML Treatment for Elthusa

XML Treatment for Elthusa raynaudii

XML Treatment for Elthusa xena

XML Treatment for Elthusa acutinasa

XML Treatment for Elthusa rotunda
